# Magneto-Ionic Physical Reservoir Computing in Perpendicularly Magnetized Heterostructures

**DOI:** 10.1021/acs.nanolett.5c03889

**Published:** 2025-10-09

**Authors:** Md Mahadi Rajib, Dhritiman Bhattacharya, Christopher J. Jensen, Gong Chen, Fahim F. Chowdhury, Shouvik Sarker, Kai Liu, Jayasimha Atulasimha

**Affiliations:** † Department of Mechanical and Nuclear Engineering, 6889 Virginia Commonwealth University, Richmond, Virginia 23284, United States; ‡ Department of Electrical and Computer Engineering, 6889 Virginia Commonwealth University, Richmond, Virginia 23284, United States; § Department of Physics, 8368 Georgetown University, Washington, D.C. 20057, United States; ∥ 96994 NIST Center for Neutron Research, Gaithersburg, Maryland 20899, United States; ⊥ Department of Electrical and Computer Engineering, Rowan University, Glassboro, New Jersey 08028, United States; ▽ National Laboratory of Solid State Microstructures, Department of Physics and Collaborative Innovation Center of Advanced Microstructures, Nanjing University, Nanjing 210093, P.R. China

**Keywords:** magneto-ionics, reservoir computing, energy-efficient, short-term memory

## Abstract

Recent progress in magneto-ionics offers exciting potential to leverage its energy efficiency for implementing physical reservoir computing (PRC). In this work, we experimentally demonstrate the classification of temporal data using a perpendicularly magnetized magneto-ionic (MI) heterostructure. The device was specifically engineered to induce nonlinear ion migration dynamics, which in turn imparted nonlinearity and short-term memory (STM) to the magnetization. These key features for enabling reservoir computing were investigated, and the role of the ion migration mechanism, along with its history-dependent influence on STM, was explained. These attributes were utilized to distinguish between sine and square waveforms within a randomly distributed set of pulses. Additionally, two important performance metrics—STM and parity check capacity —were quantified, yielding promising values of 1.44 and 2 for 24 virtual nodes, respectively, comparable to those of other state-of-the-art reservoirs. Our work paves the way for exploiting the relaxation dynamics of solid-state MI platforms and developing energy-efficient MI reservoir computing devices.

## Graphical abstract

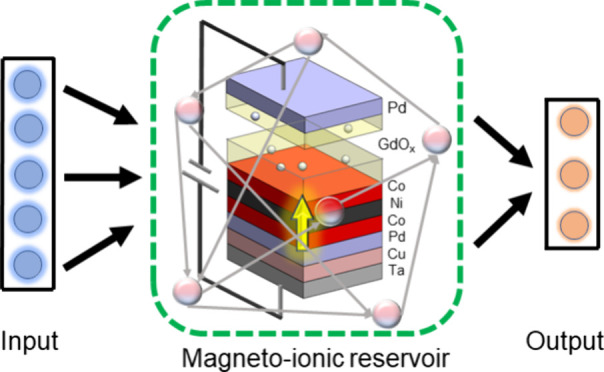

Reservoir computing is a Recurrent Neural Network (RNN)-based framework^1–7^ that processes sequential and temporal data in a simple and efficient manner. In this scheme, signals that cannot be classified within the input space can be mapped to higher dimensions by leveraging the inherent nonlinearity of the reservoir layer, resulting in linearly separable outputs. Thus, the need to train multiple connections, as is required in an RNN, is replaced by a reservoir layer. Outputs from the reservoir layer can simply be collected and multiplied by trained weights to perform classification or prediction tasks.

Central to the operation of a reservoir layer is the relaxation dynamics of a state parameter, which imparts nonlinearity and short-term memory, with past input information retained but gradually fading due to damping effects. The reservoir functionality of a physical system having a nonlinear transformation capability can be quantified with short-term memory (STM) and parity check (PC) capacities. STM capacity quantifies the reservoir’s ability to recall past inputs over time and is indicative of its memory retention for linearly decodable temporal features.^2,8^ PC capacity is a nonlinear measure that assesses the system’s ability to emulate higher-order Boolean functions, such as the parity of delayed binary inputs, and indicates the reservoir’s nonlinear processing capability.^9–11^ In a physical reservoir computer, the traditional reservoir layer is replaced with a physical system with such properties. For example, optical,^12,13^ memristive,^14,15^ mechanical,^16,17^ ionic,^18,19^ and spintronic systems^20–25^ have been demonstrated as the reservoir block of a physical reservoir computer. Among the various reservoir systems, spintronic reservoirs offer promising characteristics such as low power consumption, scalability, and CMOS compatibility.^25,26^ Various methods of controlling magnetization in spintronic devices, such as magnetic field,^27^ current,^28–32^ and electric field,^33–41^ have been demonstrated; among these, voltage-controlled methods have been shown to be the most energy efficient.^42,43^ Most studies on voltage-controlled magnetism have focused on the control of interfacial magnetism at the metal/metal oxide (MO_*x*_) interface, relying on the electric-field-induced change in the electronic structure of magnetic materials.^33–37^ However, voltage control of ionic concentration at the interface of a solid-state electrolyte and magnetic material has been shown to be highly energy-efficient,^44–48^ and the change in magnetic characteristics such as magnetic anisotropy energy can be 1–2 orders of magnitude larger.^39,44^ In particular, a perpendicularly magnetized magneto-ionic (MI) heterostructure was shown to exhibit a large VCMA coefficient of ∼5000 fJ V^–1^ m^–1^.^44,48^ Using MI effects, Namiki et al. proposed a redox-based physical reservoir utilizing the planar Hall effect and anisotropic magnetoresistance by moving Li ions at the Li_2_O–ZrO_2_–SiO_2_ (LSZO)/magnetite interface.^49^ In another work, Namiki et al. introduced iono-magnonic reservoir computing with chaotic spin wave interference manipulated by ion gating at the interface of Nafion (a proton-conducting solid-state polymer electrolyte) and Y_3_Fe_5_O_12_ (YIG).^50^ However, despite the promise of leveraging a highly energy-efficient method for controlling magnetization in a perpendicularly magnetized solid-state MI platform, MI reservoir computing has yet to be demonstrated.

Here, we experimentally demonstrate that the MI heterostructure with perpendicular magnetic anisotropy (PMA) can be utilized for the implementation of physical reservoir computing. The nonlinear dynamics of ionic migration and the corresponding change in magnetization in this platform are efficient for tasks such as temporal data classification. Particularly, we show classification of sine and square pulses from a pulse train consisting of randomly distributed sine and square pulses with 100% accuracy. Two important characteristic properties of a reservoir computer were also quantified, and promising values of 1.44 for STM and 2 for PC were found for 24 virtual nodes. For context, in a vortex-type ferromagnetic (FM) reservoir computing system, these values were found to be ∼1.5 for 250 virtual nodes.^51^ Another key advantage of MI devices is that their response time is typically in the range of milliseconds to minutes, which is similar to the time scale of many analog signals. It would be much more efficient to use MI reservoirs directly in such situations than to rely on complex electronics to convert signals to the ∼1 GHz frequencies needed for implementing reservoir computing in spintronic devices, such as spin torque nano-oscillators (STNOs).

Figure 1 illustrates the framework of the MI physical reservoir computing system. In this setup, the reservoir layer is replaced with an MI heterostructure. A pulse train consisting of randomly distributed sine and square pulses was input into the MI device as voltage pulses. These voltage pulses induced ionic movement through the solid-state GdO_*x*_ electrolyte, leading to changes in magnetization at the solid-state electrolyte/ferromagnet interface (refer to the Supporting Information S1 section for structural details). The resulting changes in magnetization were manifested in the hysteresis loops collected using a magneto-optical Kerr effect (MOKE) microscope. The coercivity values obtained from the hysteresis loops were then trained with a linear regression model for waveform classification and for quantifying STM and PC capacity (see Supporting Information S2 for details on the STM and PC capacity quantification methods).

**Figure 1:**
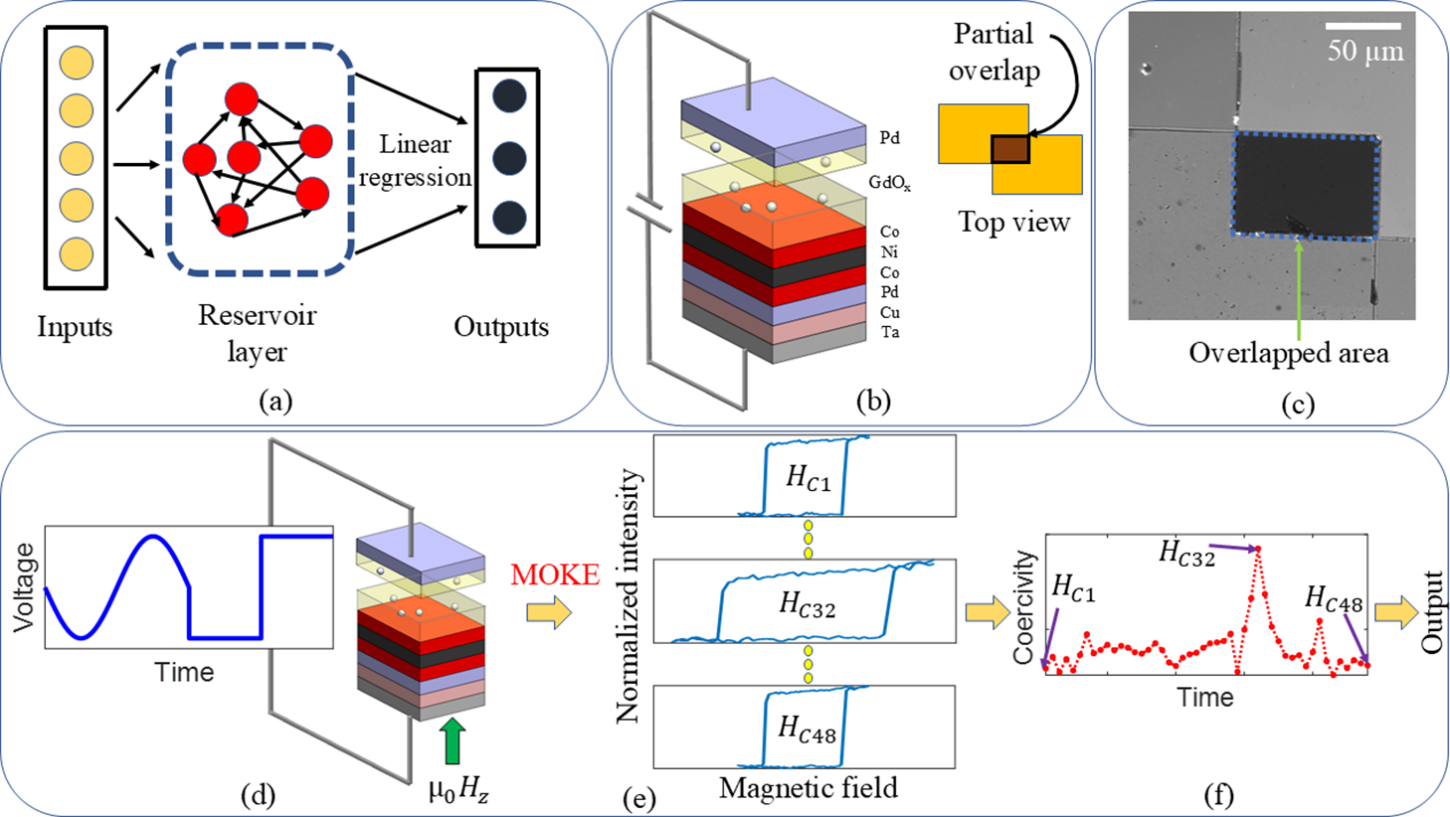
Magneto-ionic physical reservoir computing scheme. (a) Schematic of a reservoir computing system based on a recurrent neural network: Inputs are mapped to a higher dimension via the reservoir layer, and outputs from the reservoir layer are classified by using a simple linear regression model. (b) MI heterostructure as a reservoir block for physical reservoir computing: The inset depicts the partially overlapped geometry between the top and bottom electrodes. An MI PRC system is obtained by replacing the reservoir layer in (a) with the MI heterostructure. (c) MI heterostructure observed with a MOKE microscope: The area enclosed by the blue lines marks the region where changes in magnetization were manifested in hysteresis loops. (d) Schematic of the experimental setup with two input waveforms: A sine waveform followed by a square waveform from a randomly distributed pulse train was applied to the MI heterostructure via a voltage application. Magnetization changes due to the electric field across the GdO_*x*_ were measured with MOKE by sweeping an out-of-plane (OOP) field along the perpendicular direction to the heterostructure, and hysteresis loops were recorded. (e) For this combination, 48 hysteresis loops were recorded and labeled as H_c1_, H_c2_, ..., H_c48_. (f) Plot displaying 48 coercivity values for a sine pulse, followed by a square pulse.

In a solid-state MI device, an FM layer is interfaced with a solid-state electrolyte through which ions, such as oxygen,^44,52,53^ hydrogen,^46,54^ lithium,^55^ and nitrogen,^56,57^ can move upon the application of a voltage pulse. Depending on the polarity of the voltage pulse, the ions move either toward or away from the FM layer, causing a change in magnetization.^44^ Typically, these changes are nonvolatile in nature, making them useful for implementing ultralow power spintronic memory devices.^44^ However, we designed our MI device with partially overlapped geometry, as shown in Figure 1b, where a volatile change offers the ‘fading memory’ property required to implement a reservoir computer. In this geometry, the oxygen ions can move laterally in the diffusion process in addition to the vertical movement due to the electric field across the partially overlapping GdO_*x*_ electrolyte region. This is discussed in detail later. The heterostructure’s top electrode is grounded, and a voltage of ±8 V is applied at the bottom electrode, as shown in Figure 2a. Previous studies have shown that in a MO_*x*_/Co bilayer (M = Gd, Al, Mg, Ta, etc.) applying a positive voltage causes oxygen ions to migrate toward the Co layer. The resultant Co–O hybridization gives rise to the PMA.^58–61^ Conversely, a negative voltage drives the ions away, reducing the PMA and orienting the magnetization toward the in-plane direction. In our study, we observed a similar behavior: a positive voltage pulse applied to a pristine heterostructure caused oxygen ions to migrate toward the Co layer, resulting in an increase in coercivity, as shown in Figure 2a and consistent with previous reports on voltage-controlled Co/GdO_*x*_-based MI systems.^44,58,59^ A negative voltage pulse, in contrast, drove the oxygen ions away from the magnetic layer, leading to a decrease in the coercivity. As discussed earlier, these changes were volatile in nature, as illustrated in Figures 2b–2d.

**Figure 2:**
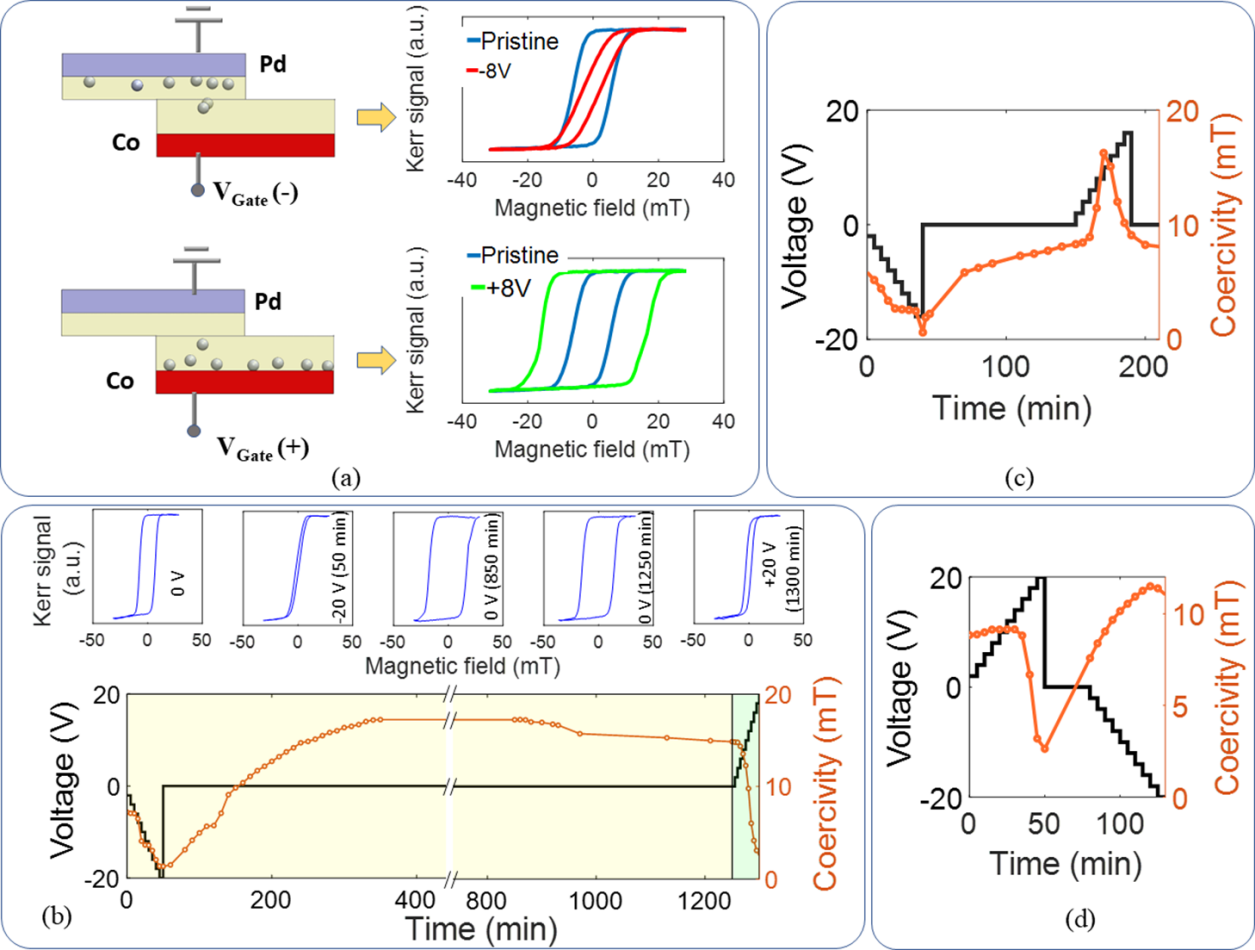
Relaxation dynamics and short-term memory. (a) Change in magnetization in a Co/Ni thin film with positive and negative voltage pulse. A negative voltage pulse drives away oxygen ions from the Co layer, reducing the coercivity, while a positive voltage pulse brings oxygen ions onto the Co surface, increasing the coercivity. (b–d) Volatile change in coercivity over time and demonstration of STM property. (Corresponding hysteresis loops are shown for 5 states in case (b).)

In the case shown in Figure 2b, as the voltage was initially decreased cumulatively from 0 to −20 V, the coercivity also decreased monotonically (hysteresis loops for the initial state and −20 V shown in the inset). We note that the voltage was incremented by 2 V, with a 5 min dwell time at each step, and it took approximately 1 min to measure each hysteresis loop. Subsequently, the −20 V voltage was removed and the coercivity was measured at different times. Interestingly, the coercivity began to increase from its minimum value upon removal of the −20 V, eventually surpassing the initial coercivity of 7.2 mT, 80 min after the voltage was withdrawn (130 min after the voltage application began on a pristine heterostructure). The coercivity then continued to rise, reaching a maximum of 17 mT at 300 min after the withdrawal of the applied voltage (350 min after the voltage application began on a pristine sample), and stabilized at this value before gradually decreasing. We note that after the withdrawal of negative voltage, oxygen ions move toward the Co layer. As the Co layer becomes oxidized and Co–O hybridization forms, PMA increases, and as a result, coercivity increases as the hysteresis loop is measured by sweeping the magnetic field in the OOP direction. As the oxidation continues beyond the optimum point, PMA decreases due to overoxidation, and coercivity also decreases, following a similar trend as reported in refs 60 and 61. While the coercivity decreased gradually at 0 V—by 2.4 mT over 400 min—the application of a positive voltage caused a more rapid reduction, with a 12.3 mT decrease observed as the voltage was cumulatively increased from 0 V to +20 V within 50 min. On a pristine heterostructure without prior gating, however, a purely positive pulse would increase coercivity, as shown in Figure 2a. These observations clearly demonstrate volatile relaxation dynamics with a pronounced history dependence on the magnetoionic response, resulting in a nonlinear input response as well as short-term memory.

The scenario shown in Figure 2c is similar to that in Figure 2b: when negative voltage pulses were applied cumulatively to the heterostructure, the coercivity decreased, and upon withdrawal of the negative pulses, the coercivity began to increase. However, in this case, a positive voltage pulse was applied before the coercivity reached its peak, in contrast to the case shown in Figure 2b, where positive voltages were applied after the coercivity had already peaked. In Figure 2b, the coercivity increased from a minimum of 1.3 mT to a maximum of 17 mT over 300 min after the withdrawal of −20 V at a rate of 0.05 mT/min at 0 V, whereas in Figure 2c, the coercivity increased from a minimum of 0.6 mT to a maximum of 16.2 mT in 130 min. In Figure 2c, the coercivity increased at a rate of 0.06 mT/min at 0 V; however, the coercivity increased at a rate of 0.4 mT/min when positive voltages were increased cumulatively from 0 V to +10 V. This cumulative application of positive pulses in Figure 2c accelerated the increase in coercivity, allowing it to reach the peak coercivity in a shorter time compared to the case in Figure 2b. After reaching the peak coercivity, further increases in the magnitude of the positive voltage up to +16 V led to a decrease in coercivity from 16.2 to 9.1 mT in 20 min, corresponding to a decrease rate of 0.4 mT/min.

Figure 2d shows the case in which positive voltage pulses were applied first. Here, the coercivity increased with the cumulatively increasing positive voltage until it reached a peak of 9.2 mT at +10 V after 25 min, as shown in Figure 2d. Beyond this peak, further increases in the positive voltage to +20 V resulted in a decrease in coercivity to 2.6 mT. Upon withdrawal of the positive voltage pulses, the coercivity began to rise again. Subsequent application of cumulative negative pulses initially increased the coercivity to 11.5 mT after 120 min at −16 V, followed by a decrease to 11.0 mT after 130 min at −20 V.

In all three cases, it is evident that the effects of positive and negative voltages were influenced by the prior history of MI changes, unlike the independent effects observed on a pristine sample. In short, the data in Figure 2 demonstrate that the MI heterostructure exhibits volatile relaxation dynamics, where the response to later pulses is governed by the history of earlier pulses, thereby imparting short-term memory properties to the system. To demonstrate these characteristics in another device architecture where electrical readout can be performed, we fabricated Hall bar devices and performed anomalous Hall effect (AHE) measurements, which is discussed in Supporting Information S3.

The origin of the relaxation dynamics of the partially overlapping geometry was studied by monitoring ion migration via optical contrast changes using a MOKE microscope.^62–64^ The partially overlapping region of the device, outlined by a white dashed line in Figure 3a, is where the electric field is primarily concentrated. To examine ion migration, optical changes were monitored not only in the partially overlapping area but also in the adjacent top and bottom electrode regions. Note that, in our material system, a darker region indicates a shortage of oxygen ions, whereas a brighter region indicates an abundance of oxygen ions.^62–64^ This is similar to the abundance and shortage of oxygen ions in a GdO_*x*_ nanowire, reflected as white and dark contrast, respectively, as observed by Kang et al. using an optical microscope.^62^

**Figure 3:**
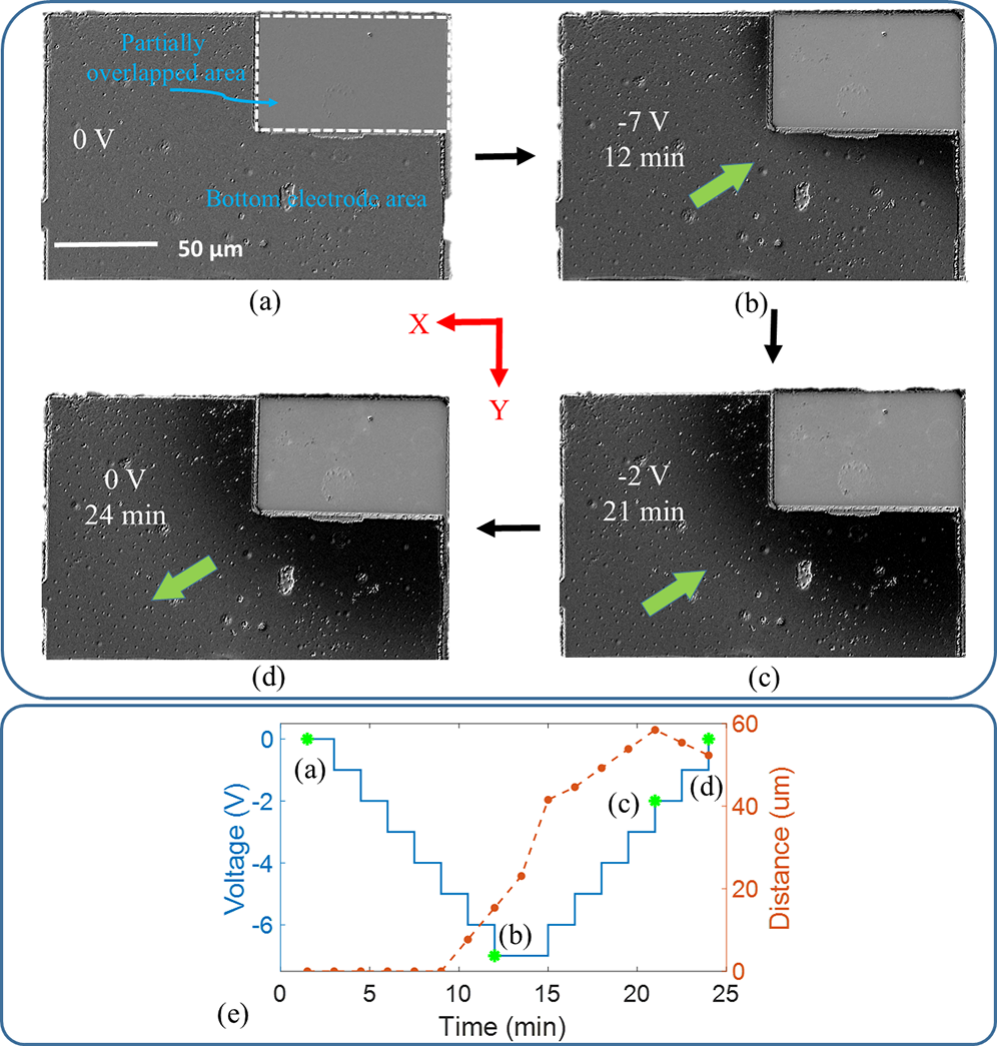
Physical explanation for short-term memory. Migration of oxygen ions upon the application of voltage pulses: (a) Pristine state with the partially overlapping region marked by a white dashed line. (b) Oxygen ions move inside the overlapping area, causing that area to become brighter due to the vertical movement of oxygen ions toward the top electrode and the diffusion of oxygen ions from the lateral bottom electrode area. (c) More oxygen ions enter the overlapping area, making it much brighter, while other areas of the lateral bottom electrode become darker. (d) The concentration of oxygen ions after the withdrawal of the voltage pulses differs from that before the application of voltage pulses in (a). (e) Voltage vs distance traveled by the oxygen ions along the *X*-axis.

To observe the migration of oxygen ions, voltage pulses were applied cumulatively. The voltage was reduced from 0 to −7 V in −1 V decrements per step, with a dwell time of 90 s at each step. Upon reaching the lowest voltage of −7 V after 12 min, the bottom electrode area adjacent to the overlapped region darkened, as shown in Figure 3b. This is because when a negative voltage was applied to the bottom electrode, oxygen ions in the overlapping region were driven toward the top electrode due to the electric field across the heterostructure, while oxygen ions from the lateral bottom electrode area diffused into the overlapping region. As a result, the bottom electrode area near the overlapping region lost oxygen and appeared darker, and the overlapping region became brighter. While the darker contrast in the bottom electrode area is readily recognizable in Figure 3b, the brighter overlapped area is not as apparent. To investigate further, we measured the intensity of the overlapped region associated with ion migration. The intensity of this region in Figure 3a is 2008 (in arbitrary units), which increases to 2295 (or 14% brighter) after the application of −7 V. Note that such a change in contrast in the bottom electrode area was not seen until −5 V was applied. This indicates that, due to the negative voltage pulses, oxygen ions were moving away from the magnetic layer, though significant lateral diffusion of ions from the adjacent bottom electrode area had not yet begun.

Subsequently, as the voltage was increased cumulatively, even at a lower negative voltage (−2 V), additional oxygen ions continued to diffuse toward the overlapping area, causing further darkening in the lateral bottom region while the overlapping region brightened (Figure 3c) with an intensity of 2301. After the negative voltages were withdrawn, the oxygen ion concentrations in both the overlapping and adjacent bottom electrode areas differed (Figure 3d) from their initial levels (Figure 3a), indicating that ion migration was not directly proportional to the applied voltage. The difference in optical contrast between the lateral bottom electrode areas in Figure 3a and Figure 3d is readily recognizable, while the difference in optical contrast in the overlapped region can be inferred from the intensities, which are 2008 and 2251 for Figure 3a and Figure 3d, respectively. Although oxygen ion migration occurs in three dimensions, measurements were taken along one axis to track the migration distance. As seen in Figure 3e, the migration distance varied nonlinearly with the applied voltage. This behavior contributed to the short-term memory property of the reservoir.

Next, we demonstrated the reservoir task. Specifically, the coercivity changes in the partially overlapping area of the device in response to randomly distributed sine and square waveforms were measured and analyzed, as illustrated in Figure 4a. A gradual increase in coercivity was observed as the pulses were successively applied, potentially caused by the irreversible increase in the ion concentration that occurred during the positive cycles of the pulses. The pulse width of the input voltage pulses was set to 36 min, and 24 hysteresis loops were measured at 90 s intervals for each waveform. Figure 4b presents a zoomed-in view of the green region from Figure 4a, showing the coercivity changes between the 27th and 30th pulses. It was observed that for square pulses the coercivity plot exhibited sharper peaks compared to sine pulses. Further analysis of the reservoir capacity of the MI system, based on how past pulses influence the magnetization response for a present pulse, is presented in the Supporting Information S4, highlighting the system’s inherent memory and nonlinearity.

**Figure 4:**
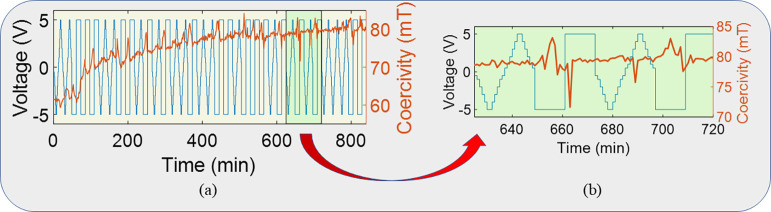
Demonstration of reservoir task. (a) Coercivity changes induced by randomly distributed sine and square voltage pulses. (b) Coercivity changes for pulses 27 to 30 are displayed in the inset (marked by green color in (a)). A distinct pattern emerges in the coercivity changes observed for sine and square pulses, with the square pulses demonstrating sharper peaks in comparison to those of the sine pulses.

These results were input into a simple linear regression model. When training was conducted on 31 input pulses and testing was performed on 4 pulses, the MI system recognized sine and square pulses with 100% accuracy. Supporting Information S5 shows testing with different numbers of test data sets. This indicated that the MI system was capable of distinguishing between sine and square waveforms within a randomly distributed set of such pulses. Additionally, the STM and PC capacities were quantified. The STM and PC capacities represent the number of data points stored for linear and nonlinear combinations of input data, respectively.^51^ By considering a delay of *D* = 3, STM and PC capacities of the MI device for 24 virtual nodes were found to be 1.44 and 2, respectively, which were comparable to those of other state-of-the-art reservoirs.^51,65–67^ We note that in a single-node reservoir, time multiplexing is utilized to mimic a reservoir,^7,68^ as was the case with the single-MI device in our experiments.

We applied randomly distributed sine and square pulses, each consisting of 24 data points, with a duration of 90 s per data point. As a result, applying 35 such pulses took approximately 21 h. Extending the number of pulses in the current system would require significantly more time.

In summary, we have demonstrated that the volatile relaxation dynamics of ionic migration in an MI device with a partially overlapping geometry give rise to short-term memory properties, making it suitable for reservoir computing. This MI reservoir is capable of performing temporal data classification tasks with a small number of training data sets, achieving 100% accuracy. Notably, our system exhibits two key characteristic properties of a reservoir computer: STM and PC capacity, with promising values of 1.44 and 2, respectively, for 24 virtual nodes.

Our work builds the foundation for Co/GdO_*x*_-based MI physical reservoir computing, and MOKE is used for the proof-of-concept demonstration. Although the use of a Hall bar or MTJ would make the measurement of coercivity or magnetization change significantly faster, the overall time scale of reservoir computing remains limited by the inherent ion migration and thus would not improve substantially. However, integrating a Hall bar or MTJ structure would significantly enhance the temporal resolution and enable a more scalable evaluation of reservoir dynamics.

The demonstrated reservoir, without optimizing for speed yet, can potentially be implemented for predicting time series such as household energy load,^22^ weather forecasting,^69^ or physiological signal analysis.^49,50^ Moreover, this time scale can be modulated—from several minutes to nanoseconds^70,71^ —by adjusting materials and control parameters such as the amplitude and duration of the applied voltage, the operating temperature,^72,73^ and the physical or chemical characteristics of the MI platform^70,71,74^ (e.g., choice of electrolyte or magnetic layer). For instance, Jeong et al. demonstrated that by inducing breakdown in the HfO_2_ gate oxide in the CoFeB/MgO/AlO_*x*_/HfO_*x*_ structure, the coercivity of the perpendicularly magnetized CoFeB could be modulated by 20% using a 20 ns gate voltage of ∼0.7 V/nm.^71^ In this way, a magneto-ionic reservoir can exhibit a wide range of temporal responses, each suitable for different types of applications. Such tunability opens the door to the development of task-adaptive MI reservoirs that match specific application requirements and is particularly valuable for energy-constrained edge computing, where optimizing both time scale and power consumption is critical. Therefore, beyond demonstrating proof-of-concept functionality, our work lays the foundation for a scalable and energy-efficient MI reservoir computing platform adaptable to a broad range of application domains.

## Supplementary Material



## Data Availability

The data that support the findings of this study are available upon reasonable request from the authors.

## Notes

Device fabrication and characterization details, classification and STM/PC quantification method, anomalous Hall Effect measurement, pulse history influence on magnetization response, classification of sine and square pulses for variable number of test data, and table on classification accuracy for variable number of testing pulses (PDF)

## Notes

Certain equipment, instruments, software, or materials are identified in this work in order to specify the experimental procedure adequately. Such identification is not intended to imply recommendation or endorsement of any product or service by NIST, nor is it intended to imply that the materials or equipment identified is necessarily the best available for the purpose.
