# Supplementary material for: Magneto-Ionic Physical Reservoir Computing in Perpendicularly Magnetized Heterostructures
Source: Nano Lett. 2025 Oct 9;25(42):15369–76. doi: 10.1021/acs.nanolett.5c03889 (PMC12550859; doi:10.1021/acs.nanolett.5c03889)
Supplement: Supplementary file 1 [file nl5c03889_si_001.pdf]

Supplementary Information

Magneto-Ionic Physical Reservoir Computing in Perpendicularly Magnetized Heterostructures

Md Mahadi Rajib[1†], Dhritiman Bhattacharya[3, 5†], Christopher J. Jensen[3,4], Gong Chen[3,6], Fahim F Chowdhury[1],

Shouvik Sarker[1], Kai Liu[3*], and Jayasimha Atulasimha[1,2,*]

[1]Department of Mechanical and Nuclear Engineering, Virginia Commonwealth University, Richmond, VA 23284, USA

[2]Department of Electrical and Computer Engineering, Virginia Commonwealth University, Richmond, VA 23284, USA

[3]Department of Physics, Georgetown University, Washington, DC 20057, USA

[4]NIST Center for Neutron Research, Gaithersburg, MD 20899, USA

[5]Department of Electrical and Computer Engineering, Rowan University, Glassboro, NJ 08028, USA

[6]National Laboratory of Solid State Microstructures, Department of Physics and Collaborative Innovation Center of Advanced Microstructures, Nanjing University, Nanjing 210093, P.R. China

S1. Device fabrication and characterization details

For fabricating the devices, a heterostructure consisting of Ta (3)/Cu (10)/Pd (3)/Co (0.7)/Ni (0.3)/Co (0.7)/GdO_x (10) (all numbers in nm), was grown on Si/SiO_2 and patterned into 300 μm squares using standard photolithography and lift-off technique. Next, GdO_x (15)/Pd (5) layers were grown using the same 300 μm square mask. This square pattern was shifted with respect to the first pattern to create the partially overlapped region, as illustrated in Figure 1b of the manuscript. All layers were sputtered using DC magnetron sputtering with a base pressure higher than 3×10^{-8} Torr. The metallic layers were sputtered with Ar working pressure of 2.5 mTorr. The GdO_x layer was reactively sputtered with a mixture of Ar (77 sccm) and O_2 (2.9 sccm) with a working pressure of 5 mTorr.

An Evico Magnetics MOKE microscope was used to observe the change in magnetization and measure the hysteresis loops. The polar mode was utilized to acquire the coercivity data. Hysteresis loops were obtained by applying an out-of-plane (OOP) field with a step size of 0.2 s, and each loop took 40 to 90 seconds to measure, depending on the range and the chosen step size of the applied magnetic field. Voltage pulses were applied to the heterostructure in situ using a Keithley 2636B source meter, while measuring the hysteresis loops with the MOKE microscope.

S2: Classification and STM/PC quantification method

We use a pulse train made up of 35 randomly distributed sine and square pulses, each with a time period of 36 minutes, to demonstrate simple temporal pattern recognition tasks. The initial 31 pulses serve as training data, while the last 4 are reserved for testing the reservoir computer. The randomly distributed sine and

square pulses (uncorrelated) are applied to the reservoir block, where they are labeled as 1 and 0, respectively, as shown in Figure S1 for the classification task:

$$p(i)=\begin{cases} 1 \text{ for sine waveform} \\ 0 \text{ for square waveform}, \end{cases} \quad (1)$$

$$p(i) = \{p_1, p_2, \dots\dots\dots, p_T, p_{T+1}, \dots., p_F\} = \{1,1,0, \dots\dots,1,1\} = \{X_T, X_P\}, \quad (2)$$

here, i denotes the index of the input pulse, where $i \in \{1,2,3, \dots\dots.T, \dots., F\}$. X_T and X_P represent the training and testing datasets, respectively:

$$X_T = \{p_1, p_2, \dots\dots\dots, p_T\},$$

$$\text{And } X_P = \{p_{T+1}, p_{T+2}, \dots\dots\dots, p_F\},$$

Here, "F" represents the total number of pulses, and "T" indicates the number of pulses used for training.

The output (coercivity) of the reservoir block is expressed as an N×F matrix:

$$H_{mn} = \begin{bmatrix} h_{11} \; h_{12} \; \dots \; h_{1F} \\ h_{21} \; h_{22} \; \dots \; h_{2F} \\ \dots. \; \dots. \; \dots \; \dots \\ h_{N1} \; h_{N2} \; \dots \; h_{NF} \end{bmatrix}; \quad (3)$$

$m \in \{1,2,3, \dots\dots\dots., N\}$ and $n \in \{1,2,3, \dots\dots\dots., F\}$,

N represents the virtual node [1], defined as $N=\frac{P}{\tau}$, where P is time period of the pulses (36 minutes), and τ is the time interval (90s) at which the output (coercivity) is measured. The concept of virtual nodes was introduced by Y. Paquot et al. [1]. By connecting virtual nodes sequentially in time and feeding them back into the nonlinear node, a specific type of recurrent neural network (RNN) architecture is created [2, 3]. In this context, virtual nodes correspond to the number of coercivity values within each time period and in our case N=24.

The weight vector is determined by:

$$W=X_T \times \text{pinv}(H_{mn}), \quad (4)$$

m ϵ {1,2,3, ..., N} and n ϵ {1,2,3, ..., T}, considering only the training data.

Here *pinv* refers to the Moore-Penrose Pseudoinverse of matrix H_{mn}

The test data are reconstructed using the weights learned during training:

$$X_{RO} = W \times H_{mn}, \quad (5)$$

m ϵ {1,2,3, ..., N} and n ϵ {T+1, T+2, ..., F} as we consider the testing data only.

The pattern recognition task evaluates the ability of the reconstructed outputs (Y_{RO}) to classify the test data (X_P).

As mentioned earlier, in addition to checking accuracy, the reservoir's performance can also be measured through its STM and PC capacities. STM capacity reflects the reservoir's ability to reconstruct past inputs based on its present outputs. The input training and testing data with delay a D ($X^{STM}_{Train,n-D}$, $X^{STM}_{Test,n-D}$) for calculating STM capacity are determined as follows:

$$X_{STM} = p(i-D) = \{X^{STM}_{Train,n-D}, X^{STM}_{Test,n-D}\}, \quad (6)$$

Conversely, PC capacity evaluates the nonlinear transformation ability of a reservoir block. The input training ($X^{PC}_{Train,n-D}$) and testing ($X^{PC}_{Test,n-D}$) data for determining PC capacity are derived using a modulo operation as follows:

$$X_{PC} = [p(i-D) + x(i-D+1) + ... + p(i)] \bmod (2) = \{X^{PC}_{Train,n-D}, X^{PC}_{Test,n-D}\}, \quad (7)$$

To evaluate STM and PC capacities, the capability of the reconstructed output to predict the test data with delay D is estimated from the following correlation coefficient [4]:

$$Cor(D) = \frac{\sum_{k=1}^{Z}(X_{Test,n-D} - < X_{Test,n-D} >)(X_{RO,n-D} - < X_{RO,n-D} >)}{\sqrt{\sum_{k=1}^{Z}(X_{Test,n-D} - < X_{Test,n-D} >)^2 \sum_{k=1}^{Z}(X_{RO,n-D} - < X_{RO,n-D} >)^2}}, \quad (8)$$

Here, $X_{Test,n-D}$ and $X_{RO,n-D}$ denote the test data and reconstructed output at a delay D, respectively, and <···> represents the mean value of "Z" number of data.

STM or PC capacity is calculated using the following equation [4]:

$$C_{STM/PC} = \sum_{D=1}^{D'} [Cor(D)]^2, \quad (9)$$

Where D' is the maximum delay which is 3 in our study. We note that the introduction of delay enables the reservoir system to classify whether the past pulse is a sine or square wave using the readout from a present pulse as shown in Figure S1.

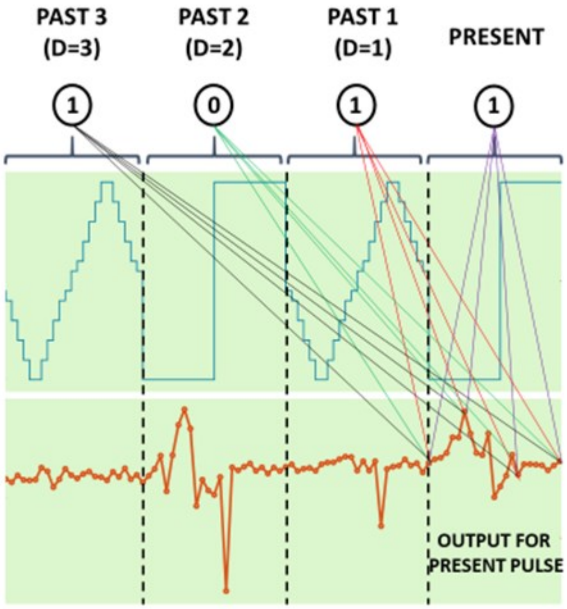

Figure S1. The output (coercivity) obtained for a present square pulse is multiplied by the computed weights to classify the past three pulses [5]. (Four pulses from a set of 35 randomly distributed pulses are shown to illustrate the classification method).

S3: Anomalous Hall Effect measurement

In the manuscript, we used MOKE for detailed magnetization tracking. To demonstrate electrical readout, we fabricated Hall bar devices as shown in Figure S2a and performed Anomalous Hall Effect Measurement (AHE). When voltage was applied, coercivity increased consistently with the applied voltage [Figure S2

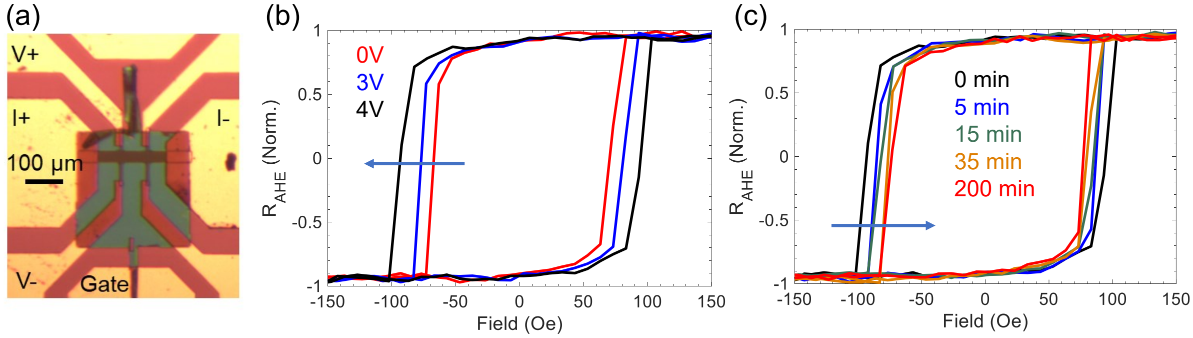

Figure S2. (a) Hall Bar device used in the experiments with dimensions of 30 µm ×180 µm while the dimensions of the gate electrode were 300 µm ×300 µm in the manuscript. The heterostructure consisted of Pd(10)/Co(0.3)/Ni(0.7)/Co(0.3)/GdO_x(40)/Pd(5), numbers are in nm. (b) Change in coercivity with increasing voltage, and (c) temporal relaxation dynamics after removal of voltage.

b]. After removal of the voltage, the coercivity decreased steadily towards the initial value. Thus, the general behavior observed in the Hall bar devices remain the same, i.e., the coercivity can be effectively modulated by applying voltage pulses and there is a history dependence of the coercivity evolution.

S4: Pulse history influences magnetization response

The memory capacity of the MI system was examined, by determining whether the current magnetization response was influenced by previous pulses, i.e., whether the magnetization response differed for various combinations of past and present pulses. Figure S3 illustrated the coercivity for the "present waveforms," which had a known past input, showing that the magnetization responses were identical for the same combination and different for different combinations of waveforms. Four possible past-present pulse combinations of two types of pulses (sine and square) were considered: square-square, sine-sine, sine-square, and square-sine, where the first pulse in each combination represents the past pulse, while the latter represents the present pulse. The response to the "present" pulse from each combination was plotted to assess how the past pulse altered the response of the present pulse. In Figures S3a, S3c, and S3b, S3d, the present pulses were square and sine, respectively. These figures display the polyfitted normalized coercivity vs timesteps. Each of the red lines in Figures S3a-3d was plotted using 24 normalized hysteresis loops (time steps), with each red line representing the output for a single pulse (either sine or square). The multiple curves in each panel correspond to individual pulses from the randomly distributed pulse train shown in Figure 4 of the manuscript. These curves are sorted based on the input 'past-present' pulse combinations, and the curves are nearly similar in each of the corresponding panels in Figure S3a-3d.

When the square waveform was applied as the present pulse, the coercivity plot exhibited a wider dome shape [see Figure S3a and Figure S3c], while for the sine pulse, the domes were narrower [see Figure S3b and Figure S3d]. However, the magnetization response varied in shape depending on the past input, although the dominant feature resulting from the present input was retained. In Figure S3a and S3c, the normalized coercivity for a square pulse as the present input is displayed, with a square pulse as the past input in case (a) and a sine pulse in case (c). Indeed, the response for the present square pulse varied in

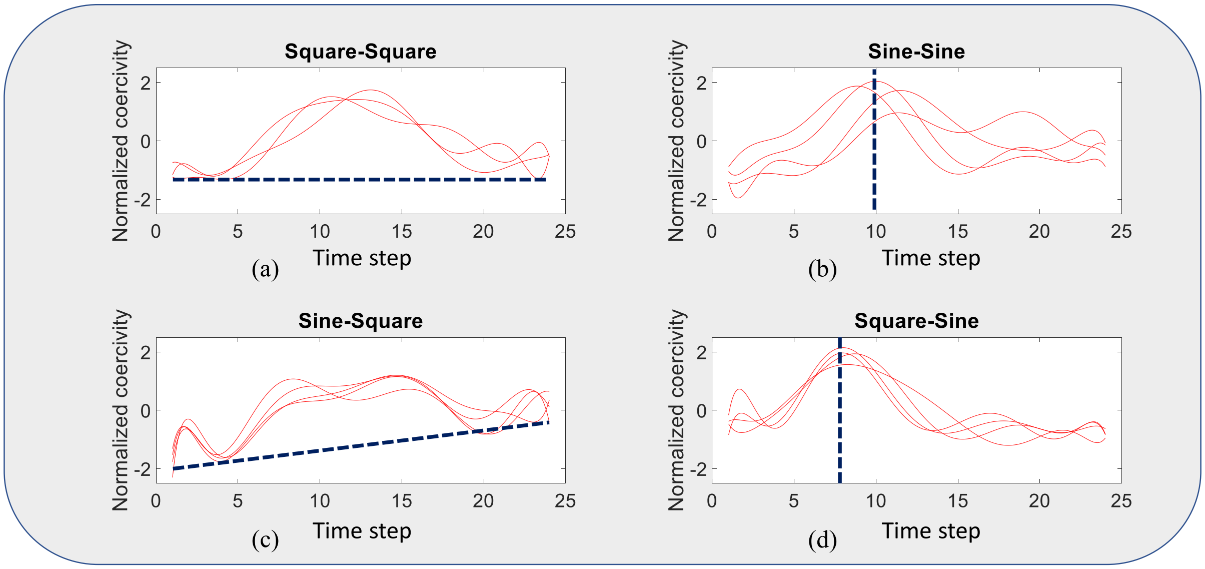

Figure S3. The normalized coercivity response for a "present" pulse from a pulse combination of (a) square-square, (b) sine-sine, (c) sine-square, and (d) square-sine.

shape depending on the past pulse. When the past pulse was square, the two ends of the dome-shaped normalized coercivity became flat (indicated by a flat dotted blue line), whereas, with a past sine pulse, the ends of the dome took on a slanted shape (indicated by a slanted blue dotted line). Despite these differences, both cases maintained the wider dome shape, which was the dominant feature caused by the present square pulse, while the orientation of the dome varied depending on the past input.

Similarly, for Figure S3b and S3d, the present pulse was sine, which resulted in a narrow dome shape as the dominant feature in both cases, with the peak of the dome occurring at the 10th time step of the pulse for past sine pulses. In contrast, the peak was observed at the 8th time step for past square pulses. These observed differences in magnetization response for varying combinations, along with the similarities for the same combination without overlap, suggested that the MI heterostructure exhibited memory and nonlinearity.

S5: Classification of sine and square pulses for variable number of test data

We use the variation in coercivity in response to pulse train consisting of 22 randomly distributed sine and square pulses as shown in Figure S4, each with a time period of 36 minutes, to demonstrate simple temporal pattern recognition tasks. The pulses are divided into training and testing sets, as shown in Table S1. Due to the long duration required to apply the pulse train, and because the device becomes saturated after many pulses as a result of asymmetric ion movement under opposite voltage polarities, we are limited to using a small number of pulses (22 pulses). Therefore, the device needs to be optimized for faster and symmetric ion dynamics to remain functional under a larger number of input voltage pulses, enabling the testing of such a reservoir on more complex tasks, such as Mackey–Glass time series prediction. As shown in Table S1, we observe that with 18 and 17 training pulses, we achieve 100% classification accuracy. However, as the number of available training pulses decreases, the classification accuracy also decreases.

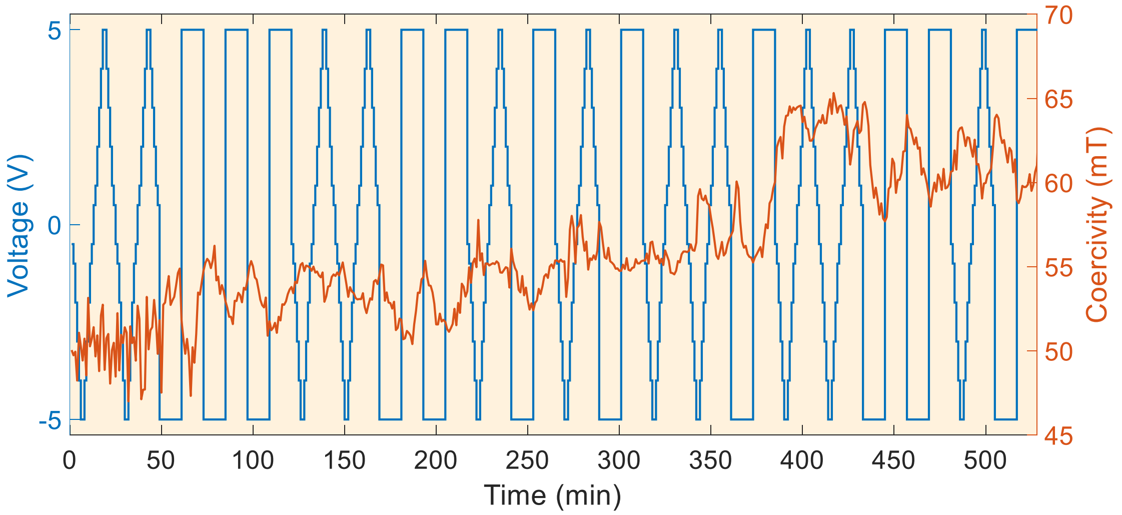

Figure S4. Coercivity variations caused by randomly distributed sine and square pulses

Table S1: Classification accuracy for variable number of testing pulses

Training	Testing	Classification accuracy (%)
18	4	100
17	5	100
16	6	83.33
15	7	85.71

References

[1] Paquot, Y.; Duport, F.; Smerieri, A.; *et al.* Optoelectronic Reservoir Computing. *Sci. Rep.* **2012**, *2*, 287. https://doi.org/10.1038/srep00287

[2] Appeltant, L.; Soriano, M.; Van der Sande, G.; *et al.* Information processing using a single dynamical node as complex system. *Nat. Commun.* **2011**, *2*, 468. https://doi.org/10.1038/ncomms1476

[3] Paquot, Y.; Dambre, J.; Schrauwen, B.; Haelterman, M.; Massar, S. Reservoir computing: a photonic neural network for information processing. *Nonlinear Optics and Applications* IV **2010**, *7728*, 58-69. SPIE. https://doi.org/10.1117/12.854050

[4] Yamaguchi, T.; Akashi, N.; Nakajima, K.; *et al.* Step-like dependence of memory function on pulse width in spintronics reservoir computing. *Sci. Rep.* **2020**, *10*, 19536. https://doi.org/10.1038/s41598-020-76142-x

[5] Pinna, D.; Bourianoff, G.; and Everschor-Sitte, K. Reservoir computing with random skyrmion textures. *Phy. Rev. App.* **2020**, 14(5), 054020. https://doi.org/10.1103/PhysRevApplied.14.054020
